# Structured Exercise During and After Incretin-Based Pharmacotherapy Discontinuation: A Narrative Review of Mechanisms, Evidence, and Prescription Guidance

**DOI:** 10.3390/healthcare14152345

**Published:** 2026-08-01

**Authors:** Zachary Zeigler, Jacob Havenar, Eddie Smith, Lillian Tang, Camryn Marthaler, Kaelyn Gardner

**Affiliations:** Department of Exercise Science and Nutrition, College of Natural Science, Grand Canyon University, Phoenix, AZ 85017, USA

**Keywords:** glucagon-like peptide-1 receptor agonist, semaglutide, tirzepatide, weight regain, resistance training, body composition, sarcopenia, cardiometabolic risk

## Abstract

**Highlights:**

**What are the main findings?**

- Incretin-based pharmacotherapy discontinuation may trigger rapid weight regain, worsening cardiometabolic risk, and disproportionate fat-preferential regain, compounded by lean mass and bone losses that accumulate during active treatment.
- Structured combined aerobic and resistance exercise, initiated concurrently with pharmacotherapy and sustained through cessation, is a biologically plausible and mechanistically coherent strategy that may attenuate the distinct physiological drivers of post-cessation rebound through pathways independent of pharmacological appetite suppression.

**What are the implications of the main findings?**

- Because lean mass losses accumulate during active treatment, clinicians considering exercise as an adjunct may wish to initiate referral concurrently with pharmacotherapy rather than deferring until cessation.
- In the absence of a randomized controlled trial, convergent mechanistic, adjacent controlled, and observational evidence provide a clinically sufficient rationale for prescribing exercise as the primary non-pharmacologic strategy in any planned or unplanned discontinuation pathway.

**Abstract:**

**Background/Objectives**: Incretin-based pharmacotherapies are the most effective non-surgical treatments for obesity to date, yet 85% of patients discontinue within the second year of real-world use. No established framework exists for managing this post-cessation transition. This narrative review evaluates the evidence for structured exercise to mitigate physiological rebound and support long-term weight management following discontinuation. **Methods**: A narrative review was conducted per Assessment of Narrative Review Articles (SANRA) guidelines using PubMed, Scopus, and Web of Science from January 2005 through to June 2026. **Results**: Post-cessation weight regain averages 5.6 kg within one year and is observationally associated with dose-dependent cardiometabolic worsening. Lean mass losses averaging 6–7 kg and reductions in bone mineral density are seen during active treatment, creating a metabolically unfavorable state at cessation compounded by fat-preferential regain. Exercise and pharmacotherapy generate fundamentally different body composition outcomes. Exercise preserves lean mass, bone density, cardiorespiratory fitness, and insulin sensitivity, while pharmacotherapy alone does not, with downstream implications for resting metabolic rate and post-cessation rebound. Controlled trial data show exercise initiated during incretin treatment is associated with significantly less weight regain, greater sustained weight loss, and maintained physical activity levels one year after medication and supervised program end. This evidence derives from a single liraglutide-based trial and may not generalize directly to semaglutide, tirzepatide, or next-generation agents. Real-world data associate exercise counseling with durable weight loss after discontinuation. **Conclusions**: No randomized controlled trial has directly evaluated exercise at pharmacotherapy cessation; conclusions are based on post-treatment extension, real-world observational, and mechanistic evidence. Combined aerobic and resistance training is a biologically plausible, low-risk adjunct to incretin-based pharmacotherapies. Resistance training, adequate dietary protein, and individualized clinical screening are likely important components pending direct evidence from post-cessation trials.

## 1. Introduction

Obesity is a chronic, relapsing disease driven by complex interactions among biological, behavioral, and environmental factors, and remains a major contributor to global cardiovascular and metabolic morbidity [1]. Obesity now affects more than one billion adults globally, with prevalence having more than doubled since 1990 [2]. Despite a longstanding emphasis on lifestyle interventions, traditional approaches combining diet, physical activity, and behavioral therapy typically yield modest weight loss of 5–10%, with substantial long-term regain [3]. Earlier generations of anti-obesity pharmacotherapies were similarly limited by modest efficacy and safety concerns, with several agents withdrawn after widespread use due to adverse effects [4].

Incretin-based pharmacotherapies mimic or potentiate the incretin hormones glucagon-like peptide-1 (GLP-1) and glucose-dependent insulinotropic polypeptide (GIP). Incretin pharmacotherapy has transformed the pharmacological management of obesity with the introduction of semaglutide, a GLP-1 receptor agonist (GLP-1RA), and tirzepatide, a dual GIP/GLP-1 receptor agonist. In Phase 3 trials, semaglutide 2.4 mg/week produced placebo-subtracted weight loss exceeding 10%, with approximately one-third of participants achieving ≥20% reduction in baseline body weight [5]. Similarly, tirzepatide, a dual GIP/GLP-1 receptor agonist, has demonstrated mean weight loss of 19.5% and 20.9% at 10 mg and 15 mg, respectively, compared with 3.1% in the placebo groups [6]. Additionally, semaglutide has been associated with reductions in blood pressure (BP), atherogenic lipids, and other cardiovascular risk factors, as well as improvements in physical function and quality of life [5,7]. These findings have positioned incretin-based therapies as the most effective non-surgical treatment for obesity to date.

However, real-world use is distinguished by high discontinuation rates. Observational data consistently show that approximately 20–50% of patients discontinue therapy within the first year, with rates exceeding 85% by year two [8,9]. These figures contrast sharply with adherence rates greater than 85% reported in clinical trials [10], highlighting the discrepancy between trail efficacy and real-world effectiveness. The reasons for discontinuation include cost and insurance barriers, gastrointestinal side effects, and limitations on medication access [11,12].

Rapid weight regain typically follows discontinuation. In the STEP 1 trial extension, semaglutide discontinuation was followed by regain of approximately two-thirds of prior weight loss at one year [13]. Similarly, in a post hoc analysis of the SURMOUNT-4 trial, 82.5% of participants who discontinued tirzepatide regained at least 25% of their prior weight loss by 52 weeks [14]. Liraglutide discontinuation has been associated with more modest regain, though estimates vary by follow-up duration [15,16,17]. This regain is accompanied by worsening cardiometabolic risk factors, including increases in waist circumference, BP, glycemia, and atherogenic lipids [16]. Recent evidence suggests that discontinuation may also be associated with increased rates of major adverse cardiovascular events (MACE), independent of changes in weight or glycemic control [17]. Furthermore, weight loss associated with incretin-based pharmacotherapy includes a substantial proportion of lean mass, often accounting for 20–40% of total weight loss [18,19]. Loss of lean mass may reduce resting metabolic rate and contribute to insulin resistance, impairing the body’s ability to maintain weight loss following cessation.

Recent narrative reviews have examined the broader relationship between GLP-1RA therapy and exercise over the treatment period, including the combined effects of these modalities on weight loss, lean mass, and cardiometabolic outcomes [20]. However, the post-discontinuation period has received substantially less attention. Critically, none of these reviews have proposed or evaluated a structured framework for exercise as a primary strategy specifically around the cessation event. A recent review comprehensively characterized the cardiometabolic consequences of GLP-1RA and tirzepatide discontinuation, including weight regain, glycemic deterioration, and the potential amplification of cardiovascular risk through weight and HbA1c cycling [21]. Strategies to preserve the benefits achieved after treatment withdrawal included structured exercise, but were not substantively addressed. Existing research has largely focused on exercise during active pharmacotherapy, but fails to address the post cessation period [22,23]. Behaviors contributing to weight maintenance post-GLP-1 therapy have not been rigorously studied.

The primary aim of this narrative review is to evaluate the role of exercise as a behavioral bridge strategy, namely, an intervention initiated during incretin-based pharmacotherapy and sustained through discontinuation, to mitigate physiological rebound and support long-term weight management. Specifically, this review synthesizes evidence on: (1) the cardiovascular, metabolic, and musculoskeletal consequences of incretin-based pharmacotherapy cessation; (2) body composition and cardiometabolic changes during pharmacotherapy and their implications for post-cessation recovery; (3) the mechanistic rationale for exercise as a behavioral bridge; (4) the evidence for structured exercise to attenuate post-cessation rebound and support long-term weight management; and (5) an evidence based exercise prescription.

## 2. Materials and Methods

This narrative review was conducted in accordance with the Scale for the Assessment of Narrative Review Articles (SANRA) guidelines [24], with attention paid to the foundational elements of rigor for narrative reviews described by Sukhera [25]. A narrative rather than systematic review format was selected because the evidence base relevant to this question spans fundamentally heterogeneous study types. These include randomized controlled trials in adjacent populations, post hoc and secondary analyses, observational cohorts, and mechanistic and preclinical studies. None of which directly test structured exercise as a primary intervention following the discontinuation of incretin-based pharmacotherapy. This heterogeneity precludes meaningful outcome pooling or the application of a formal risk-of-bias framework. The review’s central contribution is thematic organization and explicit grading of evidence (Table 1) rather than exhaustive retrieval or quantitative synthesis.

A literature search was conducted using PubMed as the primary database, with supplementary searches performed in Scopus and Web of Science. The search covered the literature published between January 2005 and June 2026; January 2005 was selected to align with the period of modern incretin-based pharmacotherapy development. An initial targeted search combined terms specific to structured exercise interventions after discontinuation of incretin-based pharmacotherapy. This search returned no directly relevant trials, confirming the evidence gap that motivates this review. The search was therefore expanded iteratively into five searches corresponding to the review’s thematic domains, using the search strings provided in Supplementary Table S1. In PubMed, these searches returned 127 records for Theme 1 (cardiometabolic consequences of cessation), 425 for Theme 2 (body composition during treatment), 735 for Theme 3 (exercise–GLP-1RA physiological interaction), and 86 for Theme 4 (post-cessation exercise as a bridge strategy). Following title and abstract screening for relevance and supplementary reference-list snowballing, 6, 13, 13, and 9 studies were retained as evidentiary sources for Themes 1–4, respectively, and are summarized in Table 1. Theme 5 (exercise prescription recommendations) drew on the established general exercise science and weight-maintenance literature. The 14 sources retained for this theme were purposively selected for their standing as foundational or guideline-level evidence rather than identified through a discrete search count. Scopus and Web of Science were searched with the same strings and used as supplements to identify additional sources not indexed in PubMed.

Studies were included if they reported on weight regain, body composition, cardiometabolic outcomes, or exercise interventions in the context of incretin-based pharmacotherapy treatment or discontinuation, or if they provided mechanistic evidence relevant to these pathways. Studies were excluded if they were not available in English, were duplicate reports of the same dataset, or did not address any of the review’s five thematic domains. Reference lists of retrieved articles were manually reviewed to identify additional relevant sources.

Evidence was prioritized in the following order: randomized controlled trials (RCTs) and meta-analyses of RCTs, followed by prospective observational studies, mechanistic/preclinical studies, and expert consensus statements. This hierarchy was also applied to assign each source in Table 1 a Level of Evidence rating (High, Moderate, Low, or Very Low), reflecting study design and methodological rigor, with ratings adjusted downward for major limitations such as non-peer-reviewed status, small sample size, substantial heterogeneity, or high risk of confounding. Preprints and the conference abstract included in this review had no peer-reviewed counterpart available at the time of the search. They are explicitly identified as such at each point of citation in the Results, with their methodological limitations discussed alongside the reported findings.

Consistent with SANRA guidance for narrative reviews, this search and selection process was not designed to be exhaustive, and no claim of complete capture of the literature is made. Rather, the search was structured to identify the studies most directly relevant to the review’s five thematic domains. Selection prioritized post-treatment extension and real-world observational evidence bearing directly on the post-cessation question where available and, otherwise, drew on the strongest available indirect or mechanistic evidence, as detailed in Table 1. Themes are presented in the order of the clinical pathway from incretin-based pharmacotherapy treatment through discontinuation to post-cessation management.

## 3. Results

### 3.1. Cardiovascular and Metabolic Consequences of Incretin-Based Pharmacotherapy Cessation

In a meta-analysis of 18 RCTs encompassing nearly 3771 participants, Tzang et al. reported a pooled mean weight regain of 5.6 kg, accompanied by significant increases in waist circumference, body mass index (BMI), systolic BP, fasting glucose, and HbA1c [18]. Participants who were followed beyond 26 weeks regained 2.9 times as much weight as those who were followed for 26 weeks or less. Semaglutide discontinuation was associated with greater regain than liraglutide. In a preprint, Budini et al. modeled the regain trajectory, estimating a plateau at approximately 76% of on-treatment weight loss, with roughly 60% of the weight loss regained by one year [26]. Similarly, in the STEP 1 trial extension, Wilding et al. followed a subset of participants off treatment for 1 year after 68 weeks of semaglutide 2.4 mg, during which both the drug and the structured lifestyle intervention (including physical activity counseling) were discontinued simultaneously [13]. Participants regained approximately two-thirds of their prior weight loss, with the proportion achieving clinically meaningful weight loss (≥5%) falling from 86.4% at treatment end to 48.2% one-year post-cessation. The authors noted that the absence of continued lifestyle support after withdrawal may have contributed to the regain trajectory.

The magnitude of cardiometabolic deterioration after cessation may be proportional to the degree of weight regain. Horn et al., in a post hoc analysis of the SURMOUNT-4 trial, stratified 308 participants who discontinued tirzepatide into four regain categories over 52 weeks [14]. Deterioration in waist circumference, systolic BP, HbA1c, non-HDL cholesterol, and fasting insulin followed a dose–response pattern across all groups. Participants who regained less than 25% of their weight loss showed no statistically significant change in waist circumference, non-HDL cholesterol, or fasting insulin relative to the end of treatment. The absence of body composition and physical activity data is a key limitation of this research.

Incretin-based pharmacotherapy discontinuation is also associated with hard cardiovascular events, though these data derive from observational cohorts rather than randomized discontinuation. Piccini et al., in a retrospective cohort study of 550 individuals with type 2 diabetes followed for five years, found that incretin-based pharmacotherapy discontinuation was independently associated with a greater than threefold increased risk of MACE in primary prevention (HR 3.40; 95% CI: 1.82–6.32) and a greater than twofold increased risk in secondary prevention (HR 2.71; 95% CI: 1.46–5.01), after multivariable adjustment [19]. Notably, changes in BMI and HbA1c were not independently associated with MACE risk in the primary prevention model, suggesting that unmeasured factors beyond weight and glycemic control may contribute to this association. Importantly, residual confounding by disease severity, access to ongoing care, adherence behavior, socioeconomic factors, or baseline cardiometabolic risk cannot be excluded and may explain some or all of this signal.

In a conference abstract, Tajerian et al. studied nearly 290,000 adults initiating injectable semaglutide or tirzepatide across more than 20,000 US clinics. Early discontinuers had higher rates of acute coronary syndrome, coronary artery disease, heart failure, and stroke compared with long-term users, and elevated risks for coronary artery disease and heart failure persisted in the post-cessation period [27]. As with the Piccini findings, this association is drawn from observational, non-randomized data and is vulnerable to confounding. Also, the reported studies in this section did not stratify outcomes by specific agent, precluding assessment of whether this cardiovascular signal differs across semaglutide, tirzepatide, or other incretin-based therapies.

### 3.2. Body Composition Changes During Incretin-Based Pharmacotherapy and Implications for Post-Cessation Recovery

A clinically significant proportion of the weight loss during incretin-based pharmacotherapy is from lean mass, including bone mineral density, rather than from fat alone [39,40]. Semaglutide 2.4 mg/week produces a mean lean mass loss of 6.9 kg over 68 weeks, representing 13.2% of baseline lean mass and approximately 40% of total weight lost; tirzepatide produced a lean mass loss of approximately 6 kg, or 10.9% of baseline lean mass, over 72 weeks [39,40]. A 10% or greater reduction in lean mass over 68–72 weeks approximates 20 years of age-related lean mass decline [40]. Following weight loss, this reduction in lean mass could be compounded by adaptive thermogenesis, a suppression of resting metabolic rate that exceeds what body composition changes alone would predict [41,42]. Lean mass loss figures should be interpreted with caution: dual-energy X-ray absorptiometry (DXA)-measured fat-free mass is roughly double the mass of skeletal muscle tissue and includes water, organs, connective tissue, and bone in addition to contractile muscle, meaning that lean mass losses during pharmacotherapy likely overstate the true magnitude of skeletal muscle and contractile protein loss [43]. Additionally, these figures describe changes in tissue mass and density; they do not by themselves establish loss of muscle strength, physical function, or frailty risk, which are distinct constructs assessed directly in Theme 3. Whether or not lean mass loss itself contributes to treatment discontinuation remains unestablished [44].

Weight regained after stopping pharmacotherapy is disproportionately composed of adipose tissue, with little recovery of lean mass and, theoretically, little restoration of energy expenditure [40]. This fat-preferential regain is often observed in the weight cycling literature and produces a ratchet effect. Each cycle deposits more fat relative to the lean mass lost, possibly worsening body composition even when overall weight returns to a prior level [45]. The hormonal basis for this drive was established by Sumithran et al., who followed participants for 62 weeks after a mean weight loss of 13.5 kg and found that appetite-regulating hormones remained significantly altered one full year after peak weight loss, with subjective hunger ratings similarly elevated throughout [46]. Van Baak and Mariman describe how adipocyte shrinkage following weight loss triggers stress responses, extracellular matrix remodeling, and reduced lipolytic capacity, all of which prime the adipose depot for restoration [47]. When pharmacotherapy is withdrawn, the appetite suppression that partially counteracted this neuroendocrine pressure is immediately lost, removing the only exogenous barrier to full fat restoration.

Rapid weight reduction may lead to bone loss through mechanical unloading, a phenomenon well documented in the literature [48,49,50]. Hansen et al., in a placebo-controlled RCT of semaglutide 1.0 mg weekly for 52 weeks in 64 predominantly postmenopausal adults at increased fracture risk, found that semaglutide produced significantly greater reductions in lumbar spine and total hip areal bone mineral density (BMD) than placebo and reduced tibial volumetric BMD and cortical thickness [28]. Bone resorption markers increased significantly in the semaglutide group, while formation markers were unchanged. At the pooled level, Kim et al. found no statistically significant change in BMD across seven RCTs [29]. This null finding contained substantial heterogeneity: most included trials used shorter-acting agents in patients with type 2 diabetes, who have characteristically elevated baseline BMD. Sensitivity analyses excluding studies of type 2 diabetes and short-acting agents showed significantly lower total hip BMD in incretin-based pharmacotherapy-treated groups. Authors reported that incretin-based pharmacotherapy significantly increased bone resorption markers across all studies.

The most direct evidence comes from the bone secondary analysis of the S-LiTE trial by Jensen et al. [30]. In 195 adults with obesity randomized to exercise, liraglutide, combination treatment, or placebo following a low-calorie diet, liraglutide alone significantly reduced hip and lumbar spine BMD compared with both exercise and placebo. Exercise alone preserved BMD at both sites relative to placebo. Critically, the combination of exercise and liraglutide, despite producing the largest weight loss of any arm, preserved BMD at both the hip and spine, indistinguishable from placebo. One proposed mechanism is that by maintaining the muscular mechanical loading and myokine signaling that stimulate bone formation, exercise effectively substitutes for the skeletal stimulus partially lost when body weight decreases [51,52].

### 3.3. Impact of Incretin-Based Pharmacotherapy Treatment on the Exercise Response

The evidence for this theme is primarily from the S-LiTE randomized controlled trial [31,43,53], conducted at the University of Copenhagen, which followed an 8-week low-calorie diet phase in which 195 adults with obesity lost a mean of 13.1 kg. Participants were then randomized for 52 weeks to moderate-to-vigorous exercise plus placebo, liraglutide 3.0 mg plus usual activity, the combination of both, or placebo alone. At 52 weeks, all active interventions achieved significantly greater weight maintenance than placebo, with the combination producing the largest effect (−9.5 kg vs. placebo; 95% CI: −13.1 to −5.9), followed by liraglutide alone (−6.8 kg) and exercise alone (−4.1 kg). Reductions in fat mass, body-fat percentage, and waist circumference followed the same hierarchical pattern, with combination values approximately twice those of the other arms. Despite producing comparable total weight loss, liraglutide alone and exercise alone generate fundamentally different lean mass outcomes. The exercise group increased lean mass by 2.1 kg over 52 weeks, while the liraglutide group showed no change [40,53]. The combination group preserved lean mass (+0.5 kg) while achieving the largest fat mass reduction in any arm. A systematic review of nine studies by Gomes Filha et al. similarly found that combining GLP-1 analogues with exercise produced approximately 5 kg greater weight loss than either intervention alone [33]. Structured exercise during liraglutide treatment also produced clinically meaningful improvements in physical functional performance and cardiorespiratory fitness that pharmacotherapy alone could not replicate [32]. In a 52-week secondary analysis of the S-LiTE trial, combined exercise and liraglutide improved stair climb performance by 8.6% and cardiorespiratory fitness by 3.0 mL/min/kg of fat-free mass relative to liraglutide alone. Exercise alone produced nearly identical improvements, while liraglutide alone had no significant effect on either measure, despite producing significant weight loss. Absolute muscle strength was maintained across all groups, including the placebo group, indicating no meaningful loss of strength during this trial, regardless of intervention. However, muscle quality (strength relative to lean mass) was preserved only in the active treatment arms and declined in the placebo group. Exercise adherence was equivalent with and without pharmacotherapy, suggesting liraglutide may not interfere with the capacity to perform exercise.

Incretin-based pharmacotherapy reduces food intake through central nervous system mechanisms that suppress appetite and slow gastric emptying [54], with no mechanism to spare skeletal muscle during energy deficit [40]. As already noted, approximately 25–40% of the weight lost during incretin-based pharmacotherapy is attributable to lean mass [20,40]. Exercise, by contrast, provides a direct mechanical and anabolic stimulus to skeletal muscle through pathways entirely independent of caloric intake; pharmacotherapy may not engage these pathways or replicate them [55,56,57,58]. The result is that two interventions that produce similar weight loss may yield fundamentally different body compositions, with possible implications for resting metabolic rate, post-cessation fat regain, and long-term metabolic resilience.

A prespecified secondary analysis of the S-LiTE trial by Sandsdal et al. examined three cardiometabolic outcomes in 130 adherent participants: metabolic syndrome severity z-score (MetS-Z), android fat percentage by DXA, and high-sensitivity C-reactive protein (hsCRP) [31]. With respect to metabolic syndrome severity, liraglutide and the combination both produced significant reductions versus placebo, moving participants into a lower cardiovascular risk category. Exercise alone did not reduce cardiovascular risk category, a pattern the authors attribute to liraglutide’s glycemic impact. All three active interventions significantly reduced android fat percentage compared with placebo, but the combination produced approximately twice the reduction in either monotherapy.

For systemic inflammation, only the combination group significantly reduced hsCRP compared with placebo, with levels approaching 1 mg/L, below the 3 mg/L threshold associated with elevated cardiovascular risk. Exercise alone did not show a significant change, likely because the preceding low-calorie diet had already substantially reduced hsCRP. Exercise was also the only intervention that maintained the large reductions in insulin resistance induced by the low-calorie diet at one year [31], a finding that is particularly significant given that insulin resistance is a possible mediator of cardiometabolic risk during weight regain [14]. The combination’s unique ability to simultaneously reduce abdominal fat, lower systemic inflammation, and preserve insulin sensitivity, while also maintaining lean mass and bone, may establish a more favorable biological state from which the post-cessation period begins.

Several outcomes documented in the S-LiTE primary analysis were exclusive to exercise-containing arms and are not achievable through pharmacotherapy-induced weight loss alone [53]. Cardiorespiratory fitness improved in both the exercise and combination groups, but not with liraglutide alone. Additionally, liraglutide alone was associated with increased resting heart rate, whereas this effect was not observed when liraglutide was combined with exercise, suggesting that exercise-induced cardiac adaptation attenuates a known pharmacological adverse effect. Exercise-containing arms also maintained improvements in general health perception and emotional well-being, outcomes not observed with liraglutide.

Nejati et al., in a meta-analysis of 16 studies encompassing 1562 participants with type 2 diabetes, demonstrated that exercise training significantly elevates endogenous GLP-1 levels, with longer-term training producing larger effects than short-term training [34]. All included studies used aerobic modalities, an evidence gap warranting dedicated investigation. The clinical magnitude of these exercise-induced GLP-1 elevations is modest relative to pharmacological doses. The finding, however, suggests that exercise engages the GLP-1 signaling axis endogenously, providing a possible physiological rationale for why the two interventions may produce effects that are more than additive when combined.

Åkerström et al. found that 10 weeks of endurance training increased the beta-cell C-peptide response to GLP-1 infusion by approximately 28 ± 43% in a small sub-study of 6 overweight, non-diabetic women, suggesting that training may improve beta-cell sensitivity to GLP-1 [35]. While this finding requires replication in adequately powered trials, it offers a mechanistic hypothesis. At the preclinical level, Wu et al. provided molecular evidence that GLP-1 signaling in skeletal muscle promotes metabolic adaptations that enhance exercise capacity via an AMPK-dependent pathway, though direct translation to humans remains to be demonstrated [56]. Exercise-induced preservation of lean mass during energy restriction is supported by controlled trials in similar populations [38,43]. Exercise-induced elevation of endogenous GLP-1 has been demonstrated in meta-analytic data, though exclusively in type 2 diabetes populations using aerobic modalities [34]. By contrast, the attenuation of neuroendocrine adaptations following incretin withdrawal, and GLP-1 receptor upregulation in the post-cessation context have not been directly tested in this population and should be understood as theoretical extrapolations.

### 3.4. Exercise as a Behavioral Bridge Strategy Post-Cessation

The National Weight Control Registry, comprising over 10,000 individuals who maintained substantial long-term weight loss, consistently identifies regular physical activity as one of the most common behavioral characteristics of successful maintainers. Most members report approximately one hour of moderate-intensity activity per day [59,60,61]. Ramage et al., in a systematic review of 67 weight loss maintenance interventions, found that physical activity was a component of 88% of successful programs [62]. Reviews of RCTs examining exercise after weight reduction report mixed results, with only a minority showing statistically significant differences in weight regain between exercise-assigned and control groups, a finding primarily attributed to adherence failures [59,63]. However, when RCT participants who actually exercised are compared with those who did not, a consistent dose–response relationship with weight maintenance emerges [60]. The American College of Sports Medicine (ACSM) position stand identifies 200–300 min per week to prevent weight regain after successful weight loss, substantially above the 150 min per week recommended for general health maintenance [64].

The neuroendocrine milieu following incretin-based pharmacotherapy withdrawal differs fundamentally from the post-weight-loss state produced by dietary restriction alone. Avogaro and Fadini describe withdrawal of exogenous incretin hormones as abruptly removing the pharmacological suppression of appetite while endogenous GLP-1 secretion returns to its intrinsically insufficient baseline, producing rebound hyperphagia, reduced energy expenditure, impaired postprandial glucose control, and preferential fat restoration [65]. Exercise may mitigate this biology through two distinct pathways. The first is the energy flux framework described by Foright et al. [60]. Weight-reduced individuals exist in a metabolically unfavorable ‘low flux’ state characterized by low energy intake, even lower expenditure, and a large energy gap between appetite and requirements. Exercise may shift patients toward a higher flux state, where both intake and expenditure are elevated but more closely balanced, narrowing the gap that drives relapse. At cessation, appetite rebounds sharply while energy expenditure remains suppressed, widening the energy gap at the point of greatest vulnerability. Exercise, by shifting the patient toward higher flux at this transition, could narrow the energy gap. The application of the energy flux framework to the post-cessation transition remains a theoretical extrapolation not yet directly tested in this population.

The second pathway operates through lean mass preservation. Resistance exercise during incretin-based pharmacotherapy may preserve lean mass [40]. Thus, the resulting maintenance of resting metabolic rate is physiologically plausible and well established in other populations, but it has not been directly measured following incretin-based pharmacotherapy and should be considered an inferred possibility. The post-treatment extension of the S-LiTE trial is the most closely related study to the question of whether exercise influences outcomes after the discontinuation of incretin-based pharmacotherapy [53]. Following the 52-week active treatment period, all participants entered a 1-year off-treatment phase in which both pharmacotherapy and the supervised exercise program were withdrawn, with no structured lifestyle support provided to any arm. At post-treatment assessment, the combination group maintained a 5.1 kg greater weight reduction than liraglutide alone (95% CI −10.0 to −0.2 kg; *p* = 0.040), with a greater reduction in body fat percentage (−2.3 percentage points; *p* = 0.026). Weight regain during the off-treatment year was 6.0 kg greater with liraglutide alone than with supervised exercise alone (95% CI, 2.1–10.0 kg). A substantially larger proportion of those who had exercised sustained at least 10% weight loss one year after treatment termination, compared with liraglutide alone (OR 4.2; 95% CI 1.6–10.8). Participants previously randomized to exercise engaged in significantly more moderate- and vigorous-intensity physical activity in the week before post-treatment assessment than those who received liraglutide alone, suggesting that the supervised program may have established durable exercise habits [53]. As the only trial to directly assess this question, this study’s findings are specific to liraglutide and have not yet been replicated with semaglutide or tirzepatide, agents associated with larger on-treatment lean mass losses.

In a preprint that has not yet undergone full peer review, Murugadoss et al. reportthe only available real-world human data associating exercise counseling with post-cessation weight outcomes [37]. These findings are preliminary and require confirmation in peer-reviewed, prospective studies before they can be considered established. In the subset of patients with clinician-documented discontinuation, exercise counseling was documented significantly more frequently among those with durable weight loss than among those who regained (26.2% vs. 14.7%; *p* = 0.04), while diet counseling rates were nearly identical between groups (22.3% vs. 23.5%; *p* = 0.83). Exercise may be a biologically plausible and worthy candidate for prospective testing during the post-cessation transition.

### 3.5. Exercise Prescription Guidance

The recommendations that follow are extrapolated from adjacent populations (e.g., diet-induced weight loss, GLP-1RA-plus-exercise trials), not from direct trial data in the post-cessation population, and should be understood as a starting framework for clinical judgment and future trial design. These recommendations are drawn exclusively from peer-reviewed sources; no preprint included elsewhere in this review informs the prescription guidance below. DXA-measured lean mass losses averaging 6–7 kg and measurable reductions in bone mineral density provide justification for prioritizing resistance training in the exercise prescription for this population [39,40]. Supervised resistance training programs for more than 10 weeks attenuate lean mass loss and improve strength relative to a diet-only comparator in adults with obesity, and they represent a possible countermeasure to the lean mass losses documented during incretin-based pharmacotherapy [39]. Table 2 summarizes this framework across the treatment timeline to support individualized clinical decision-making. Table 3 adapts this framework for populations commonly encountered among patients receiving incretin-based pharmacotherapy, drawing on current condition-specific consensus guidelines.

However, resistance training alone may not be the ideal program. Villareal et al. directly compared aerobic, resistance, and combined training during caloric restriction in 160 obese older adults and found that combined training produced the greatest improvement in physical function, the greatest fat mass reduction, and preserved lean mass nearly as well as resistance training alone, while additionally delivering the cardiovascular fitness benefits that resistance training alone cannot provide [38]. Aerobic training alone produced substantially greater lean mass loss than the combined group (−2.7 kg vs. −1.7 kg), despite similar total weight loss. Mechanick et al. similarly recommend that physical activity programs for this population include both aerobic and resistance components [39].

For the aerobic component, current guidance recommends at least 200 min per week of moderate-to-vigorous physical activity to prevent weight regain following successful weight loss. This figure reflects total physical activity volume; moderate-intensity resistance training could contribute to this threshold, reinforcing rather than competing with the combined-modality prescription [71]. For the post-cessation population entering a period of active appetite rebound, the upper end of this range may be a reasonable target, based on the rationale that appetite rebound increases the energy gap that exercise is intended to narrow. Moderate-intensity continuous training at 65–85% of heart rate reserve, as used in the LITOE trial, provides a practical, well-tolerated intensity in populations not on incretin therapy [38]. Tolerability during active pharmacotherapy may require intensity modification due to gastrointestinal side effects, as discussed below.

For the resistance component, the ACSM recommends muscle-strengthening activities targeting all major muscle groups on at least 2 non-consecutive days per week [58]. For strength development, heavier loads of ≥80% one-repetition maximum with 2–3 sets produce superior strength gains. For lean mass preservation, volume is the critical driver, with ≥10 sets per muscle group per week at moderate-to-heavy loads [58]. Lower volumes may be sufficient for preservation in previously untrained beginners. Training to momentary muscle failure is not required to achieve these adaptations and is potentially inadvisable in deconditioned older adults due to elevated cardiovascular stress and injury risk from compromised form. A working target of approximately 2–3 repetitions in reserve is the appropriate intensity guide. Mechanick et al. note that muscle loss during incretin-based pharmacotherapy may affect some muscle groups more than others, depending on sex and age, reinforcing the need for individualized rather than generic resistance protocols [39].

Locatelli et al. recommend initiating resistance training at the start of incretin therapy [40]. The clinical rationale is that lean mass lost during the first weeks and months of treatment may not be recovered. The S-LiTE post-treatment data suggest that exercise habits formed during the treatment period may persist after medication cessation and are associated with less weight regain in the off-treatment year [36]. The treatment period could therefore be used not only to preserve lean mass but to habituate patients to regular exercise as a behavioral pattern that carries forward through discontinuation.

#### 3.5.1. Implementation Considerations: Safety, Individualization, and Progression

Given the prevalence of obesity-related comorbidities in this population, including hypertension, type 2 diabetes, cardiovascular disease, osteoarthritis, and obstructive sleep apnea, pre-exercise screening for unaddressed cardiovascular, metabolic, and musculoskeletal risk factors is warranted before initiating an exercise program [64,69]. Screening need not be a barrier to participation; current guidance favors a pragmatic approach that identifies clear contraindications and the need for medical clearance without unnecessarily restricting access to exercise, which carries greater benefit than risk for most individuals with obesity [64].

Gastrointestinal symptoms such as nausea, delayed gastric emptying, and altered appetite are among the most reported adverse effects of incretin-based pharmacotherapy and contributing factors to discontinuation. Multidisciplinary clinical guidance for managing GLP-1RA-associated gastrointestinal adverse events recommends smaller, more frequent meals, adequate hydration, and avoiding high-fat or high-volume meals [72]. By extension, these strategies reasonably inform recommendations for exercise timing, though no trial has directly tested GI symptom management protocols specifically in the context of exercise during or after incretin-based pharmacotherapy. Patients should not be advised to exercise on an empty stomach if appetite suppression has led to inconsistent eating patterns, since low energy availability can impair exercise tolerance and recovery [73]. Fueling strategies should be individualized in coordination with the protein and overall caloric intake guidance discussed in the following section.

Resistance training progression should begin conservatively, particularly for patients who are deconditioned, sedentary, or new to structured exercise. An initial phase of lighter loads and lower volume, with progression toward target volume and intensity over several weeks as tolerance and technique allow, reduces injury risk and improves adherence compared with the immediate prescription of target-level training [58]. Individualization should account for baseline fitness, comorbidity burden, age- and sex-related differences in muscle group vulnerability to lean mass loss, and patient-reported tolerance, rather than applying a uniform protocol across this heterogeneous population [39]. Where feasible, supervision by a qualified exercise professional, physical therapist, or appropriately trained clinical staff is preferable to a fully self-directed program. Supervision supports correct technique and safety during progression and may lead to increased adherence [36].

Adherence, rather than prescription specificity, may be the primary determinant of whether exercise functions as an effective bridge strategy. Dropout from initiated exercise programs occurs in approximately half of participants within 6 to 12 months, even when substantial behavioral and environmental support is provided [60]. Baseline physical activity level and access to exercise facilities, equipment, or safe outdoor space should therefore inform program design. Patients with low baseline activity or limited facility access may benefit from home-based or minimal-equipment protocols rather than programs assuming gym access, and progression targets should be individualized according to these constraints.

Delivery mode may also influence long-term adherence. Supervised resistance training programs attenuate lean mass loss and improve strength relative to diet-only or unsupervised comparators [39]. Digital and AI-driven coaching platforms represent a scalable alternative where in-person supervision is unavailable. In a randomized trial conducted in a non-GLP-1RA population with prediabetes, overweight, or obesity, an AI-driven lifestyle intervention was similar to human coaching for weight loss and glycemic outcomes and produced higher program initiation (93.4% vs. 82.7%) and completion (63.9% vs. 50.3%) rates [74]. Group-based care models have an established evidence base in general obesity and diabetes care, with demonstrated improvements in dietary habits, glycemic control, and modest weight reduction compared with individual visits [75]. Whether these models improve adherence specifically to exercise-based recommendations in this population has not been directly studied.

#### 3.5.2. Protein Co-Prescription as an Adjunct to Exercise

Dietary protein intake is an essential adjunct to exercise prescription in this population and should be addressed concurrently. Lean mass preservation during caloric restriction requires both adequate protein intake and resistance exercise stimulus [39]. The 2025–2030 Dietary Guidelines for Americans have increased emphasis on protein, suggesting that 1.2–1.6 g/kg body weight/day is an optimal intake level [76]. This range aligns closely with the European Society for Clinical Nutrition and Metabolism (ESPEN) Expert Group consensus of Deutz et al., which recommends 1.0–1.2 g/kg/day for healthy older adults, rising to 1.2–1.5 g/kg/day for those with illness or malnutrition risk [77]. A practical protein target of 1.2–1.6 g/kg/day is supported by both frameworks for the post-incretin population, with active monitoring warranted given associated appetite suppression and food aversion. Oral nutritional supplementation may be considered if dietary intake is insufficient. The LITOE trial used 1 g/kg/day as a dietary floor across all exercise groups, representing a conservative lower bound by ESPEN standards [38]. There is no direct trial evidence testing a specific protein target in the post-incretin-based pharmacotherapy context, and no current guideline recommends supplementation beyond individual adequacy [39].

Dietary adherence to this protein target may itself be constrained by cost. Protein-rich diets have been shown to cost significantly more per day than standard diets in other populations, a barrier that may disproportionately affect socioeconomically disadvantaged patients [78]. These targets require individualization in patients with chronic kidney disease (particularly eGFR < 30 mL/min/1.73 m^2^) or advanced liver disease, for whom protein prescription should be undertaken in consultation with the treating renal or hepatology specialist rather than applied uniformly [75]. Referral to a registered dietitian is recommended to individualize protein targets, food sources, and distribution across meals for patients with these or other conditions affecting nutritional needs.

#### 3.5.3. Special Populations: Older Adults with Sarcopenic Obesity

Older adults with sarcopenic obesity may represent the highest-risk subgroup for incretin-based pharmacotherapy-accelerated lean mass and bone loss. These patients have the greatest potential to benefit from weight loss, but also the greatest vulnerability to lean-mass loss and bone loss. For exercise prescription in this population, Chen and Batsis emphasize resistance training as a key strategy for preserving muscle mass in sarcopenic obesity [79], while established exercise guidelines recommend programs beginning with moderate loads (∼60–70% 1RM, 8–12 repetitions) and progressing in volume, intensity, and movement velocity over time [58]. The addition of balance training is important given the risk of falls and therefore should be included in programming. Villareal et al. found that resistance training specifically prevented the total hip BMD loss produced by aerobic training alone [38]. For older adults with sarcopenic obesity, functional improvement and preservation of physical independence are established co-primary targets alongside weight reduction, as reflected in current clinical guidelines [58,79]. Modifications for chronic kidney disease [68], established cardiovascular disease [69], frailty [67], type 2 diabetes [66], and osteoarthritis [70] are summarized in Table 3. These populations are inadequately represented in both the incretin and post-cessation exercise bodies of literature, and all recommendations should be applied with particular caution pending dedicated evidence. Physical therapist involvement is recommended where mobility or fall risk is a limiting factor. Higher protein intakes (1.2–1.5 g/kg/day) appear safe in individuals with normal renal function and are recommended in older adults to preserve muscle mass [77], whereas in more advanced chronic kidney disease (e.g., GFR <30 mL/min/1.73m^2^), lower protein intakes (∼0.6–0.8 g/kg/day) are typically recommended based on nephrology guidelines [80].

## 4. Discussion

Incretin-based pharmacotherapy discontinuation rates of up to 50% within the first year of therapy mean clinicians are routinely managing the post-cessation period without trial-derived guidance [8,9]. No RCT has examined structured exercise as a primary intervention following discontinuation of incretin-based pharmacotherapy. This review synthesizes mechanistically related data to support exercise as a biologically plausible, low-risk, and research-worthy strategy during and after cessation of pharmacotherapy. It must be noted that lean mass loss, discontinuation patterns, and cardiovascular outcomes differ among liraglutide, semaglutide, and tirzepatide and may influence the extent of exercise needed. A full comparative pharmacology analysis is beyond the scope of this review; readers are directed to the existing comparative effectiveness literature for agent-level detail [15,16,23,39].

The treatment period itself, rather than the cessation event, is the theoretical window for exercise intervention. As detailed in Theme 2, lean mass losses of 6–7 kg accumulate during active pharmacotherapy and are not always recovered after cessation. Additionally, weight regained post-discontinuation is disproportionately fat, progressively worsening body composition even when total weight returns to prior levels. The S-LiTE post-treatment data provide the strongest available support for early initiation, albeit from a single liraglutide-based trial. Exercise habits formed during the supervised treatment period persisted into the off-treatment year, and both the exercise-alone and combination arms showed significantly attenuated regain compared with pharmacotherapy alone [36]. Thus, if exercise is to be considered, referral should be initiated concurrently with pharmacotherapy rather than deferred until the point of cessation.

As noted, the behavioral bridge framework has been theorized mainly from the S-LiTE trial, which used liraglutide. Caution is warranted when extrapolating to other compounds. Next-generation agents under investigation, including triple agonists such as retatrutide, amylin analogue/GLP-1RA combinations such as cagrilintide-semaglutide (CagriSema), and novel combination approaches, may produce substantially greater magnitudes of weight loss and lean mass loss. The withdrawal physiology of these next-generation agents has not yet been characterized in long-term trials. If the post-cessation energy gap, lean mass deficit, and neuroendocrine rebound are proportional to on-treatment efficacy, as available evidence suggests [16,18], next-generation agents may amplify rather than reduce the physiological case for exercise as a bridge strategy.

Weight regain following incretin-based pharmacotherapy discontinuation is not driven by physiology alone. A substantial body of evidence links adherence to psychiatric comorbidities, including depression, anxiety, and disordered eating patterns, as well as environmental and socioeconomic factors [81]. As discussed in Section 3.5, these same barriers may directly impede initiation of, and adherence to, the exercise- and nutrition-based strategies proposed in this review. Low exercise adherence in weight-loss-maintenance populations is well documented, with dropout from initiated exercise programs occurring in approximately half of participants within 6 to 12 months even when substantial behavioral, psychological, and environmental support is provided [60]. A behavioral bridge strategy that depends on sustained exercise and dietary engagement should be considered alongside, rather than instead of, attention to psychological comorbidity, socioeconomic access, and behavioral counseling.

### 4.1. Limitations

This review has several limitations that should inform the interpretation of its conclusions. Most critically, the evidentiary case for exercise as a behavioral bridge rests disproportionately on the S-LiTE trial and its secondary and post-treatment analyses, conducted by a single investigative group in Denmark. The two sources most relevant to the post-cessation question in Table 1 [36,37] occupy distinct and limited evidentiary categories: post-treatment extension evidence from this one trial, and real-world observational evidence from an unreviewed retrospective cohort study relying on electronic health record documentation of exercise counseling rather than an objectively measured exercise behavior. Neither should be considered direct evidence in the sense of a controlled trial testing exercise as an intervention after discontinuation. No independent group has replicated the S-LiTE post-treatment finding in a different population, setting, or pharmacological agent. The S-LiTE trial enrolled adults with obesity but without diabetes (mean age approximately 40 years) in Denmark, a population that may differ from the real-world discontinuation population. Additionally, the S-LiTE trial used liraglutide; contemporary agents such as semaglutide 2.4 mg and tirzepatide produce larger lean mass losses [40], so the post-cessation deficit may be more severe than the S-LiTE data reflect. Furthermore, the mechanistic finding by Åkerström et al. [35] that exercise improves beta-cell sensitivity to GLP-1, was based on only 6 participants and requires replication. Also, older adults with sarcopenic obesity remain substantially underrepresented in both the GLP-1RA and exercise bodies of literature [79], despite being the subgroup at greatest risk from pharmacologically accelerated lean mass losses and bone loss. The recommendation to prioritize resistance training for lean mass preservation, while well-supported in the general weight-loss literature, has not been directly validated in a population undergoing incretin-based pharmacotherapy. A further limitation concerns extrapolating the exercise-maintenance literature to the post-incretin-discontinuation population. The weight-maintenance exercise literature was derived from populations who lost weight through caloric restriction or behavioral intervention, not pharmacotherapy. Divergent results may be observed after termination of incretin therapy.

### 4.2. Future Direction

A randomized controlled trial of supervised combined aerobic and resistance exercise, initiated at or before pharmacotherapy cessation, with primary endpoints of weight regain, body composition, and cardiovascular outcomes at 12 and 24 months post-cessation, represents the most urgent research priority in this area. Such a trial should be powered to examine older adults with sarcopenic obesity as a pre-specified subgroup [79] and should include objective physical activity monitoring to test whether behavioral carry-forward mediates outcomes [36,79]. Because discontinuation frequently occurs without planned clinical support [37,65], future work should also include behavioral support components to address adherence barriers.

### 4.3. Conclusions

Discontinuation of incretin-based pharmacotherapy is common and clinically consequential. The physiological state that follows cessation is often characterized by rebound hyperphagia, weight regain, and cardiometabolic deterioration. Thus, if exercise is to be considered, it could be initiated before or at the time of pharmacological cessation. This review proposes conceptualizing exercise as a biologically plausible, low-risk, and research-worthy strategy to preserve lean mass and establish durable habits, sustained through the cessation event to counter the physiological pressures that drive relapses. This remains a promising conceptual framework, not an established clinical standard, and requires validation through adequately powered randomized clinical trials. Until such trials are conducted, exercise as a behavioral bridge during and after incretin-based pharmacotherapy discontinuation should be understood precisely as what the available evidence supports: a low-risk, mechanistically coherent adjunct that merits both clinical consideration and urgent investigative priority.

## Figures and Tables

**Table 1:** Summary of Key Evidentiary Sources on Exercise, Incretin-Based Pharmacotherapy, and Post-Cessation Outcomes.

Study	Design	Level of Evidence	Population	Agent	Exercise Component	Discontinuation Assessed?	Main Outcome(s)	Relevance to Post-Cessation Question	Key Limitation
Tzang et al. (2025) [18]	Systematic review & meta-analysis, 18 RCTs	Moderate	3771 participants	GLP-1RAs (mixed agents)	None	Yes	Pooled mean regain 5.6 kg for semaglutide, 4.29 kg for liraglutide after cessation; greater regain with longer follow-up (>26 wks: 7.31 kg vs. ≤26 wks: 2.51 kg); cardiometabolic markers worsen proportionally with degree of regain	Indirect	No body composition or physical activity data in pooled studies; high heterogeneity across trials
Budini et al. (2025) (preprint) [26]	Systematic review + nonlinear meta-regression	Very Low	Pooled trial populations	GLP-1RAs (mixed)	None	Yes—models regain trajectory	Regain plateaus at ~76% of on-treatment loss; ~60% of lost weight regained at 1 year post-cessation	Indirect	Preprint, not peer-reviewed; modeling assumptions unverified independently
Wilding et al. (2022) (STEP 1 extension) [13]	RCT extension, 1 yr off-treatment follow-up	Moderate	1961 randomized; ~300 followed off-treatment (representative subset across 5 countries)	Semaglutide 2.4 mg	None—both drug AND structured lifestyle support (incl. physical activity counseling) withdrawn simultaneously at week 68	Yes	~2/3 of weight loss regained at 1 yr; proportion maintaining ≥5% loss fell from 86.4% (week 68) to 48.2% (week 120); weight remained 5.6% below baseline at week 120; cardiometabolic benefits partially reversed	Indirect—documents the ‘no bridge’ comparator scenario.	No continued exercise/activity intervention post-cessation; lifestyle support also withdrawn simultaneously; relatively small off-treatment subset; no body composition data during off-treatment phase
Horn et al. (2026) (SURMOUNT-4 post hoc) [14]	Post hoc analysis of RCT withdrawal trial	Moderate	308 participants who achieved ≥10% weight reduction during 36-wk tirzepatide lead-in, then randomized to placebo	Tirzepatide (10 or 15 mg)	None (continued 500 kcal/day deficit + ≥150 min/week PA counseling during placebo phase)	Yes	82.5% regained ≥25% of initial weight reduction within 1 year; greater weight regain associated with greater reversal of cardiometabolic improvements; those with <25% regain preserved most cardiometabolic benefits	Indirect—dose–response between regain magnitude and cardiometabolic deterioration established	Post hoc analysis; no body composition data; no objective physical activity or dietary measures to correlate with outcomes
Piccini et al. (2023) [19]	Retrospective cohort, 5 yr follow-up	Low	550 adults with type 2 diabetes	GLP-1RAs (mixed)	None	Yes	Discontinuation independently associated with HR 3.40 (95% CI 1.82–6.32) for MACE in primary prevention; HR 2.71 (95% CI 1.46–5.01) in secondary prevention after multivariable adjustment; BMI and HbA1c changes not independently associated with MACE in primary prevention model	Indirect—observational association, not causal evidence that discontinuation directly causes MACE	Observational design; residual confounding by disease severity, adherence behavior, access to care, socioeconomic factors, and baseline cardiometabolic risk cannot be excluded
Tajerian et al. (2025) (conference abstract) [27]	Retrospective cohort	Very Low	~290,000 adults initiating injectable semaglutide or tirzepatide across >20,000 US clinics	Semaglutide/tirzepatide	None	Yes	Early discontinuers had higher rates of acute coronary syndrome, coronary artery disease, heart failure, and stroke during treatment period vs. long-term users; elevated risks for CAD and heart failure persisting after discontinuation	Indirect—observational, no exercise variable; vulnerable to confounding by indication	Abstract-level reporting; methodological detail limited; risk of confounding by indication (sicker patients more likely to discontinue)
Hansen et al. (2024) [28]	Placebo-controlled RCT	Moderate	64 postmenopausal adults at increased fracture risk	Semaglutide 1.0 mg (lower than current obesity dose)	None	No (on-treatment)	Greater BMD loss and bone resorption marker increase vs. placebo; effect on bone remodeling markers apparent	Indirect—on-treatment bone effect; no cessation or exercise variable	Small sample (*n* = 64); lower dose than current obesity regimens (2.4 mg); postmenopausal women only, limiting generalizability
Kim et al. (2024) [29]	Meta-analysis, 7 RCTs	Low	Mixed populations, mostly type 2 diabetes	GLP-1RAs (mixed, mostly shorter-acting agents)	None	No (on-treatment)	No significant pooled BMD change overall; signal of BMD loss emerges after excluding T2D subgroup and short-acting agents; heterogeneity substantial across subgroups	Indirect—on-treatment; heterogeneous populations	Very high heterogeneity; null result sensitive to subgroup exclusions; mostly T2D/short-acting agents not representative of current obesity-dose long-acting GLP-1RAs
Jensen, Sørensen et al. (2024) (S-LiTE bone secondary analysis) ^a^ [30]	Prespecified secondary analysis of RCT	Moderate	195 adults with obesity (no diabetes), BMI 32–43 kg/m^2^	Liraglutide 3.0 mg	Exercise vs. liraglutide vs. combination vs. placebo (52 wks)	No (on-treatment)	Liraglutide alone significantly reduced hip and lumbar spine BMD vs. placebo and exercise; exercise alone preserved BMD at both sites; combination (largest weight loss) also preserved BMD, indistinguishable from placebo	Indirect—on-treatment; directly tests exercise’s bone-protective role alongside pharmacotherapy	Liraglutide is an older, lower-efficacy agent; effect magnitude may differ for semaglutide/tirzepatide; no post-cessation bone data
Sandsdal et al. (2023) (S-LiTE cardiometabolic secondary analysis) ^a^ [31]	Prespecified secondary analysis of RCT	Moderate	130 adherent participants, S-LiTE trial	Liraglutide 3.0 mg	Exercise vs. liraglutide vs. combination vs. placebo	No (on-treatment)	Combination uniquely reduced hsCRP; sustained insulin sensitivity improvement across treatment year; exercise independently improved cardiorespiratory fitness markers	Indirect—on-treatment cardiometabolic data; exercise-specific benefits demonstrated alongside pharmacotherapy	Adherent-only subgroup may not generalize; liraglutide-based; single-center Danish cohort
Jensen, Fiorenza et al. (2026) (S-LiTE physical fitness secondary analysis) ^a^ [32]	Prespecified secondary analysis of RCT	Moderate	193 adults with obesity (no diabetes), BMI 32–43 kg/m^2^	Liraglutide 3.0 mg	Exercise vs. liraglutide vs. combination vs. placebo; median 108 min/wk MVPA achieved; 2.65 sessions/wk	No (on-treatment)	Combined exercise + liraglutide improved stair climb performance by 8.6% and VO_2_ peak by 3.0 mL/min/kg FFM vs. liraglutide alone; exercise alone produced similar fitness gains; liraglutide alone had no effect on functional performance or cardiorespiratory fitness despite significant weight loss; absolute muscle strength preserved across all active groups; dose–response between exercise volume and fitness gains confirmed	Indirect	Liraglutide-based (older agent); no post-cessation fitness data; muscle-strengthening component was circuit training, not progressive resistance—likely suboptimal for hypertrophy/strength gains; generalizability limited to adults without diabetes aged 18–65
Gomes Filha et al. (2025) [33]	Systematic review, 9 studies	Low	Adults with obesity	GLP-1 analogues (mixed)	Mixed exercise interventions	No (on-treatment)	~5 kg greater weight loss with GLP-1 + exercise vs. either intervention alone; synergistic effect on fat mass reduction	Indirect—on-treatment combination effects only	Heterogeneous exercise protocols and GLP-1 agents across included studies; no post-cessation data; limited body composition detail
Nejati et al. (2022) [34]	Meta-analysis, 16 studies (1562 participants)	Moderate	Adults with type 2 diabetes	None—examines exercise effect on endogenous GLP-1 secretion	Aerobic training (varied protocols)	No	Exercise training raises endogenous GLP-1; larger effect with longer duration and higher intensity training; effect size moderated by training volume	Indirect (mechanistic)—no pharmacotherapy or cessation context	All included studies used aerobic modalities only; T2D population not directly representative of obesity-only post-cessation population
Åkerström et al. (2022) [35]	Small RCT/mechanistic substudy	Very Low	6 participants	None—examines training effect on GLP-1 receptor responsiveness	Endurance training, 10 weeks	No	~28% increase in beta-cell C-peptide response to GLP-1 infusion after training, suggesting exercise upregulates GLP-1 receptor responsiveness	Indirect (mechanistic)—preliminary, very small sample	Very small sample (*n* = 6); single finding, requires independent replication before clinical inference is warranted
Jensen, Blond et al. (2024) (S-LiTE post-treatment extension) ^a^ [36]	RCT post-treatment extension, 1 yr off-treatment follow-up	Moderate	109/166 eligible participants attended post-treatment assessment (66% of completers); adults with obesity, no diabetes, mean age ~43 yrs, single-center Copenhagen	Liraglutide 3.0 mg (discontinued at week 52, start of extension)	Exercise vs. liraglutide vs. combination vs. placebo (during prior treatment phase; no supervised intervention during off-treatment year)	Yes—week 52–104	Combination/exercise arms: −5.1 kg body weight (95% CI −10.0 to −0.2; *p* = 0.040) vs. liraglutide alone at 1 yr post-cessation; −2.3–point body fat (95% CI −4.3 to −0.3; *p* = 0.026); OR 4.2 (95% CI 1.6–10.8) for maintaining ≥10% weight loss vs. liraglutide alone; weight regain 6.0 kg (95% CI 2.1–10.0) larger after liraglutide vs. exercise in off-treatment year	Post-treatment extension evidence—only RCT-derived evidence that exercise habits formed during pharmacotherapy treatment are associated with attenuated regain in the post-cessation year	Liraglutide-based (older, lower-efficacy agent); no supervised exercise during the off-treatment year itself; self-selected attendance at post-treatment visit (66% return rate, likely survivor bias toward better responders); single-center Danish cohort not representative of real-world discontinuation population
Murugadoss et al. (2026) (preprint) [37]	Retrospective real-world cohort	Very Low	Subset of patients with clinician-documented GLP-1RA discontinuation (semaglutide/tirzepatide);	Semaglutide/tirzepatide	Exercise counseling—documented in EHR at point of discontinuation (not directly measured or objectively verified)	Yes—discontinuers only	Exercise counseling documented significantly more frequently among patients with durable weight-loss maintenance vs. those who regained (26.2% vs. 14.7%; *p* = 0.04)	Real-world observational evidence—only real-world human data associating exercise specifically with outcomes after contemporary incretin-based pharmacotherapy discontinuation	Preprint, not peer-reviewed; EHR counseling documentation is a proxy for exercise behavior, not a measured behavioral or outcome variable; retrospective design; confounding by indication (patients counseled on exercise may systematically differ from those not counseled); cannot establish causality
Villareal et al. (2017) (LITOE trial) [38]	RCT	High	160 obese older adults (≥65 yrs) undergoing caloric restriction; no pharmacotherapy	None (diet-based weight loss; no GLP-1RA/incretin therapy involved)	Aerobic vs. resistance vs. combined training; balance training (10 min/session); ≥1 g/kg/day protein floor; 26-week supervised program	No—no pharmacotherapy involved; diet-induced weight loss only	Combined training produced greatest physical function gain (6 min walk, stair-climb, balance) and fat loss; resistance training specifically prevented total hip BMD loss seen with aerobic-only training; near-equivalent lean mass preservation to resistance alone; combined superior for functional outcomes	Indirect—informs exercise prescription design for the post-cessation population	No GLP-1RA/incretin therapy involved; older adult population (≥65 yrs) may not generalize to younger post-cessation patients; diet-induced weight loss produces smaller lean mass deficits than contemporary pharmacotherapy

Post-treatment extension evidence refers to data derived from continued follow-up of a randomized trial’s own treatment arms after the intervention period ends. Real-world observational evidence refers to retrospective or registry-based data collected outside a controlled trial setting, which may rely on proxy measures (e.g., documented counseling) rather than objectively measured behavior. Indirect/mechanistic evidence concerns related but distinct populations or pathways. Level of Evidence (High/Moderate/Low/Very Low) reflects study design and methodological rigor per the evidence hierarchy described in Methods. Narrative review articles are not included in this table, as they do not constitute independent evidentiary sources. RCT = randomized controlled trial; BMD = bone mineral density; MACE = major adverse cardiovascular events; T2D = type 2 diabetes; GLP-1RA = glucagon-like peptide-1 receptor agonist; hsCRP = high-sensitivity C-reactive protein; FFM = fat-free mass; MVPA = moderate-to-vigorous physical activity; EHR = electronic health record; CAD = coronary artery disease; OR = odds ratio; HR = hazard ratio; PA = physical activity; LOE = Level of Evidence. ^a^ All S-LiTE-derived analyses were conducted by the same Copenhagen research group and share the same underlying trial population.

**Table 2:** Proposed Framework for Individualized Exercise Prescription Across the Incretin-Based Pharmacotherapy Treatment Timeline.

Treatment Phase	Aerobic Component	Resistance Component	Key Individualization Considerations
Initiation/Early Treatment (first weeks, dose titration)	Begin at tolerated intensity; defer high-volume aerobic work if GI symptoms are prominent during titration	Initiate resistance training without delay at conservative volume/load; progress gradually as tolerance allows	Screen for cardiovascular/metabolic/musculoskeletal contraindications; assess baseline fitness and facility access to select supervised vs. home-based protocol
Active Treatment (steady-state dosing)	Minimum: 75 min/week vigorous-intensity or interval activity. Progressive target: ≥200 min/week moderate-to-vigorous activity as tolerated	Minimum: 2 sessions/week, all major muscle groups, 2–3 sets/exercise. Progressive target: ≥2 non-consecutive days/week; ≥10 sets/muscle group/week (higher-volume tier) for lean mass preservation	Individualize load/intensity by age- and sex-related muscle group vulnerability; adjust timing around GI symptom pattern; prioritize habit formation during this phase
Peri-Cessation (planned discontinuation approaching)	Maintain established aerobic volume; do not taper exercise alongside medication taper	Maintain established resistance frequency and volume	Reinforce that exercise habits formed here are hypothesized to carry forward post-cessation; counsel patient on anticipated appetite rebound
Post-Cessation	Minimum: maintain established volume. Higher-volume target (selected patients): upper end of 200 min/week range reasonable given appetite-rebound energy gap, pending direct trial confirmation	Continue established resistance program at target or higher-volume tier (~10 sets/muscle group/week); do not discontinue alongside medication	Reassess baseline activity/facility access, as post-cessation circumstances may differ from treatment-phase circumstances; monitor for regain-associated disengagement

This framework is a proposed, extrapolated synthesis intended to guide clinical judgment and future trial design; it is not derived from direct trial data in the post-cessation population and should not be interpreted as a validated clinical algorithm. Minimum and progressive-target values are drawn from current aerobic and resistance training guidelines (see Table 3); higher-volume targets reflect this review’s synthesis for selected patients. Special population modifications (e.g., sarcopenic obesity in older adults) are detailed in Table 3 and should be layered onto this framework rather than treated as a separate protocol.

**Table 3:** Population-Specific Modifications to the General Exercise Prescription Framework.

Population	Modification to General Tiered Prescription	Source
General adult (baseline reference)	Minimum feasible dose: 75 min/week vigorous-intensity or interval aerobic activity, or resistance training 2 sessions/week (all major muscle groups, 2–3 sets/exercise). Progressive target: 150 min/week moderate-to-vigorous aerobic activity plus resistance training 2–3 sessions/week. Higher-volume target (selected patients): ~10 sets/muscle group/week for hypertrophy-focused lean mass preservation.	ADA (2026) [66] Currier et al. (2026) [58]
Younger adults/higher baseline fitness	The minimum threshold may be met with shorter, vigorous-intensity or interval sessions (as little as 75 min/week) rather than the standard 150 min/week target.	ADA (2026) [66]
Older adults/frailty	Multicomponent programming (resistance, balance, and mobility training) is prioritized over aerobic volume alone; intensity and progression should be individualized to functional capacity rather than applying the general tiered targets uniformly. Progressive resistance training is considered a cornerstone intervention.	Izquierdo et al. (2025) [67]
Type 2 diabetes	Resistance training on 2–3 nonconsecutive days/week is specifically recommended. Muscle-strengthening exercise is explicitly noted as likely beneficial for maintaining lean body mass in patients treated with obesity pharmacotherapy.	ADA (2026) [66]
Chronic kidney disease	≥150 min/week moderate-intensity activity is reasonable, or to a level compatible with the patient’s cardiovascular and physical tolerance; prescription should be individualized to functional capacity, with protein targets adjusted by eGFR, and undertaken in consultation with the treating nephrology specialist for advanced disease.	Navaneethan et al. (2025) [68]
Established cardiovascular disease	Formal cardiac clearance is recommended prior to progression to vigorous-intensity aerobic or resistance training; progression should proceed more conservatively than the general tiered targets pending clearance.	American College of Sports Medicine (2025) [69]
Osteoarthritis (knee/hip)	Land-based exercise, including resistance training, is considered core, first-line treatment rather than an optional adjunct; delivery (modality, supervision, and progression) should be individualized per international consensus recommendations.	Holden et al. (2023) [70]

This table adapts the general tiered prescription in Table 2 for populations commonly encountered among patients receiving incretin-based pharmacotherapy. It draws on current, condition-specific consensus guidelines rather than direct evidence in the post-cessation population, and modifications should be applied in conjunction with, not in place of, individualized clinical judgment and, where indicated, specialist consultation.

## Data Availability

No new data were created or analyzed in this study. Data sharing is not applicable to this article.

## Supplementary Materials

The following supporting information can be downloaded at https://www.mdpi.com/article/10.3390/healthcare14152345/s1. Supplementary Table S1. Search Strings by Thematic Domain.

## Abbreviations

The following abbreviations are used in this manuscript:

ACSM	American College of Sports Medicine
BMD	Bone mineral density
BMI	Body mass index
DXA	Dual-energy X-ray absorptiometry
GIP	Glucose-dependent insulinotropic polypeptide
GLP-1	Glucagon-like peptide-1
GLP-1RA	Glucagon-like peptide-1 receptor agonist
HDL	High-density lipoprotein
hsCRP	High-sensitivity C-reactive protein
MACE	Major adverse cardiovascular events
MetS-Z	Metabolic syndrome severity z-score
RCT	Randomized controlled trial
SANRA	Scale for the Assessment of Narrative Review Articles
