# Supplementary material for: Structured Exercise During and After Incretin-Based Pharmacotherapy Discontinuation: A Narrative Review of Mechanisms, Evidence, and Prescription Guidance
Source: Healthcare (Basel). 2026 Aug 1;14(15):2345. doi: 10.3390/healthcare14152345 (PMC13464992; doi:10.3390/healthcare14152345)
Supplement: Supplementary file 1 [file healthcare-14-02345-s001.zip › healthcare-4432133-supplementary.pdf]

Supplementary Table S1. Search Strings by Thematic Domain.

All searches were conducted in PubMed with a January 2005–June 2026 date filter applied. Equivalent searches were run in Scopus and Web of Science using the same strings, adapted to each database's field-tag syntax, and used supplementally to identify additional sources not indexed in PubMed. Theme 5 was not bounded by a discrete search count; see Methods for rationale.

Theme	Search string (PubMed syntax)
1. Cardiometabolic consequences of cessation	("glucagon-like peptide-1 receptor agonist" OR "GLP-1RA" OR semaglutide OR tirzepatide OR liraglutide) AND (discontinuation OR cessation OR withdrawal) AND ("cardiovascular risk" OR "cardiometabolic outcomes" OR "weight regain" OR "weight cycling")
2. Body composition change during treatment	("glucagon-like peptide-1 receptor agonist" OR "GLP-1RA" OR semaglutide OR tirzepatide OR liraglutide) AND ("body composition" OR "lean mass" OR "skeletal muscle")
3. Exercise–GLP-1RA physiological interaction	("glucagon-like peptide-1 receptor agonist" OR "GLP-1RA" OR semaglutide OR tirzepatide OR liraglutide) AND (exercise OR "physical activity" OR "resistance training" OR "aerobic exercise")
4. Post-cessation exercise as a bridge strategy	("glucagon-like peptide-1 receptor agonist" OR "GLP-1RA" OR semaglutide OR tirzepatide OR liraglutide) AND (discontinuation OR cessation OR withdrawal) AND (exercise OR "physical activity" OR "resistance training" OR "aerobic exercise")
5. Exercise prescription evidence	*Not applicable—sources purposively selected from foundational/guideline-level exercise-science and weight-maintenance literature rather than identified through a discrete database search (see Methods).*
